# Boosting the Solar Water Oxidation Performance of Fe_2_O_3_ Photoanode via Embedding Laser‐Generated Pt Nanocrystals

**DOI:** 10.1002/smsc.202300318

**Published:** 2024-02-27

**Authors:** Fan Li, Jie Jian, Shiyuan Wang, Lichao Jia, Hongqiang Wang

**Affiliations:** ^1^ School of Physics and Information Technology Shaanxi Normal University Xi'an Shaanxi 710119 China; ^2^ School of Materials Science and Engineering Northwestern Polytechnical University Xi'an Shaanxi 710072 China; ^3^ Chongqing Innovation Center of Northwestern Polytechnical University Northwestern Polytechnical University Chongqing 401135 China; ^4^ School of Materials Science and Engineering Shaanxi Normal University Xi'an Shaanxi 710119 China

**Keywords:** composite Fe_2_O_3_@Pt, nanocrystals embedding, nanoporous α‐Fe_2_O_3_, photoanodes, photoelectrochemical water splitting

## Abstract

α‐Fe_2_O_3_ with suitable band structure, good chemical stability, and easy preparation, is a potential photoanode material. However, the key to enhance the performance of α‐Fe_2_O_3_ photoanode is to improve the transport characteristics of bulk carriers. It is expected to form a Schottky barrier to improve the carrier separation efficiency by embedding metal nanoparticles into the matrix, but the process is still challenging. Herein, a strategy of forming the Schottky barrier is shown to improve bulk carrier transport dynamics by embedding laser‐generated Pt nanocrystals in α‐Fe_2_O_3_ photoanode, which achieves photocurrent densities of up to 1.16 mA cm^−2^ at 1.23 V_RHE_ (from original 0.21 mA cm^−2^). This work provides another way to promote the carrier transfer and separation of α‐Fe_2_O_3_, which is of great significance to improve the photoelectrochemical water splitting performance.

## Graphical abstract

A strategy of embedding laser‐generated Pt nanocrystals in Fe_2_O_3_ photoanode is developed to enhance driving force of carrier separation and transport for boosting its photoelectrochemical (PEC) performance. The prepared composite Fe_2_O_3_@Pt photoanodes with local nano‐heterointerfaces exhibit excellent photovoltage and photocurrent density, which provide new insight and direction into the design of high‐performance PEC photoelectrodes.

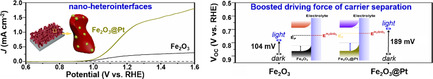

## 1 Introduction

Photoelectrochemical (PEC) water splitting is the key technology in response to the rapid deterioration of the global climate and the shortage of fossil energy,^[^ ^1^, ^2^, ^3^ ^]^ which is the ideal technology for conversion and storage of solar energy. Photoelectrode is the core part of the PEC device,^[^ ^4^, ^5^, ^6^ ^]^ its performance directly determines the solar‐to‐hydrogen conversion efficiency.^[^ ^7^, ^8^, ^9^ ^]^ Among various photoelectrode materials, inorganic semiconductor photocatalysts have obtained extensive research for their sufficient earth abundance and stability in water.^[^ ^10^, ^11^, ^12^, ^13^ ^]^ In which, α‐Fe_2_O_3_ (“α‐” is omitted thereafter) has been widely used photoanode because of its narrow bandgap, good chemical stability in alkaline solution, low cost, and environment‐friendly.^[^ ^14^, ^15^, ^16^ ^]^ However, due to its sluggish carrier transport in bulk and slow carrier transfer at semiconductor–liquid junctions (SCLJs), the PEC efficiency of α‐Fe_2_O_3_ is far from the practical application under AM 1.5G solar simulated light.^[^ ^17^, ^18^, ^19^ ^]^

Element doping can change the energy band structure of Fe_2_O_3_,^[^ ^20^, ^21^, ^22^, ^23^, ^24^, ^25^ ^]^ increase the carrier concentration and carrier lifetime, improve the electrical conductivity, and increase the electric field at the electrode/electrolyte interface to inhibit the carrier recombination.^[^ ^15^, ^26^, ^27^ ^]^ Noticeably, doping with elements Pt can improve the charge transfer characteristics in the bulk of the Fe_2_O_3_, and thus resulted in a record‐breaking performance at that time.^[^ ^28^ ^]^ To promote the carrier separation and external transport, coating ultrathin oxide/nonoxide overlayer on Fe_2_O_3_ is the best choice,^[^ ^25^, ^29^, ^30^, ^31^ ^]^ based on which the obtained Fe_2_O_3_/TiO_2_/FeOOH photoanode by integration of the three modifications achieved a remarkable PEC performance.^[^ ^14^ ^]^ Similarly, other oxide/nonoxide materials such as Cu:NiO_*x*_,^[^ ^32^ ^]^ Al_2_O_3_,^[^ ^33^ ^]^ CDots,^[^ ^34^, ^35^ ^]^ and metal‐organic framework‐derived p‐Cu_2_O^[^ ^23^ ^]^ were successfully employed to decorate the Fe_2_O_3_ surface to enhance the carrier transfer.

However, most of the previous works were a specific modification to address a single specific issue, and the excellent photoelectrode results from the superimposition of multiple modification strategies. Here, inspired by the success of Pt element doping in Fe_2_O_3_ photoanode, and the strategy of embedding laser‐generated nanocrystals in material matrix proposed by Wang's group,^[^ ^36^, ^37^, ^38^, ^39^, ^40^ ^]^ we demonstrate a strategy of embedding laser‐generated Pt nanocrystals in Fe_2_O_3_ photoanode to promote the carrier transport in bulk and carrier transfer at SCLJs for boosting its PEC performance, leading to the improved photocurrent density 1.16 mA cm^−2^ at 1.23 V_RHE_ (from original 0.21 mA cm^−2^), and significantly increase of bulk charge separation efficiency about 2 times at 1.23 V_RHE_.

## 2 Results

### 2.1 Synthesis of Pt Nanocrystals via Laser Irradiation

**Figure 1:**
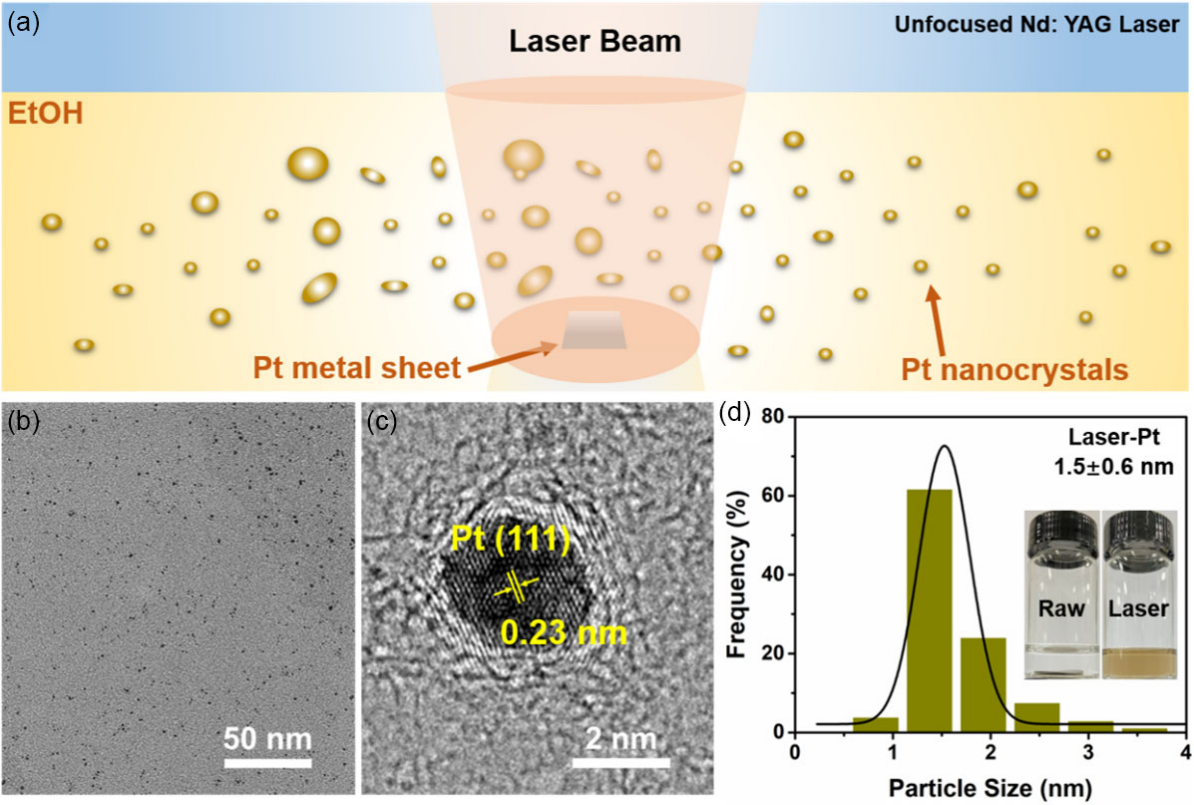
a) Schematic illustration of Pt nanocrystals generated by PLIL. b) TEM image and c) HRTEM image of Pt nanocrystals. d) Size distribution of Pt nanocrystals (insert: photographs of Pt solution before and after PLIL).

**Figure** **1**a schematically illustrates the preparation of ligand‐free Pt nanocrystals (Pt NCs) via the unfocused pulsed laser irradiation in liquid (PLIL). The Pt metal sheet was immersed in ethanol, and then the mixture was irradiated with the laser flux of 1.0 J pulse^−1^ cm^−2^ for 5 min. Subsequently, a yellow‐brown transparent colloidal solution was obtained (Figure 1d–inset), and the concentration of Pt NCs was around 0.28 mg mL^−1^ (Table S1, Supporting Information). The transmission electron microscopy (TEM) image shows that the size of the particles generated by the laser is less than 2 nm (Figure 1b). Furthermore, high‐resolution TEM (HRTEM) shows marked lattice fringes (0.23 nm) corresponding to the Pt (111) crystal planes. Particle size statistics (Figure 1d) shows that the size of Pt nanocrystals is almost less than 2 nm, mainly distributed in 1–3 nm. Compared with the traditional wet chemical synthesis, PLIL technology can prepare nanocrystals in situ in solution without any ligands, which is conducive to the transmission of electrons.^[^ ^41^, ^42^ ^]^

### 2.2 Embedding Laser‐Generated Pt Nanocrystals in Fe_2_O_3_ Photoanode

In this article, the preparation process of Fe_2_O_3_ film is in accordance with the spin‐coating method previously reported. Figure S1a, Supporting Information, describes the preparation process of Fe_2_O_3_ film. In short, a layer of FeOOH film is deposited on  fluorine‐doped tin oxide (FTO) substrate by spin‐coating, and then FeOOH is transformed into Fe_2_O_3_. To construct Fe_2_O_3_@Pt thin films, Pt NCs were prepared in situ in ethanol, and then the Pt NCs colloidal solution was mixed with the Fe(NO_3_)_3_ precursor to form the Fe_2_O_3_@Pt precursor solution. The specific manufacturing process is shown in Figure S1b, Supporting Information. Then, we embed the Pt NCs with different concentrations in Fe_2_O_3_ films, which are denoted as Fe_2_O_3_@Pt‐1, Fe_2_O_3_@Pt‐2, Fe_2_O_3_@Pt‐3, Fe_2_O_3_@Pt‐4, and Fe_2_O_3_@Pt‐5. All films were prepared on FTO substrates followed by an annealing step at 500 and 800 °C in air, respectively.

**Figure 2:**
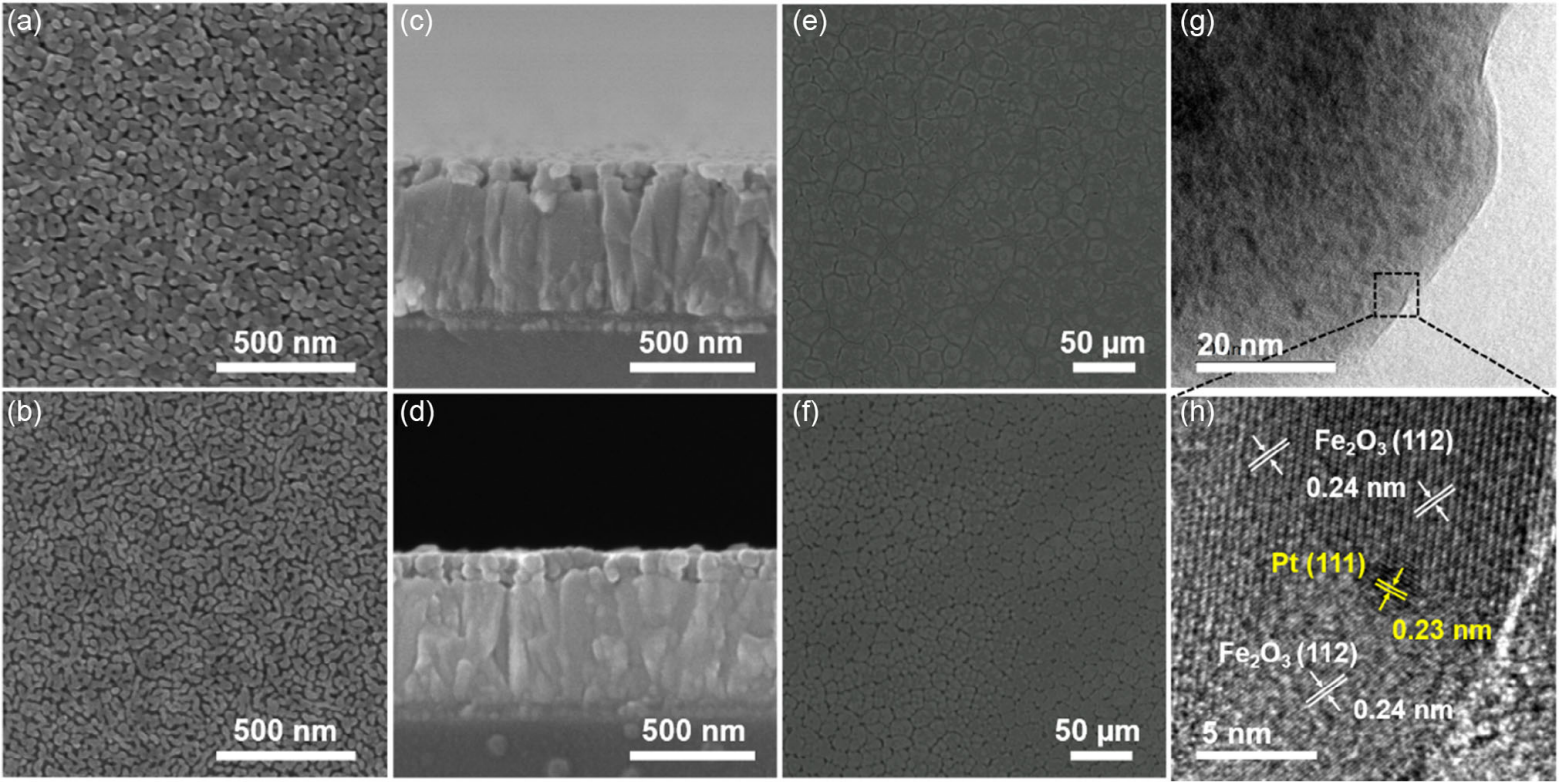
a) Surface SEM images of Fe_2_O_3_ and b) Fe_2_O_3_@Pt‐2 films. c) Cross‐section SEM images of Fe_2_O_3_ and d) Fe_2_O_3_@Pt‐2 films. e) Optical microscopies of the Fe_2_O_3_ precursor and f) Fe_2_O_3_@Pt‐2 precursor. g) TEM and h) HRTEM images of Fe_2_O_3_@Pt‐2 film.

The prepared Fe_2_O_3_ films show worm‐like nanoporous structure (**Figure** **2**a), and the thickness of the film is about 100 nm (Figure 2c). TEM images show that the crystallinity of the particles in the Fe_2_O_3_ film is good (Figure S2a, Supporting Information). The HRTEM (Figure S2b, Supporting Information) image shows the row spacing of 0.22 and 0.24 nm, which matches the (200) plane and (112) of α‐Fe_2_O_3_, respectively.^[^ ^39^ ^]^ After embedding Pt NCs, the particle size of the films decreased significantly (Figure 2b). With the increase of the concentration of Pt NCs, this trend becomes more obvious, which can be seen from the scanning electron microscopy (SEM) images of the films prepared with different concentrations of Pt NCs (Figure S3a,d, Supporting Information). However, too many Pt NCs would lead to an inhomogeneous film (Figure S3e,f, Supporting Information). There is no significant change in the thickness of the film (Figure 2c,d) because the concentration of the precursor solution does not change and the limited concentration of Pt NCs has little effect on the properties of the solution. Further, the optical microscopies of the Fe_2_O_3_ precursor in Figure 2e,f imply that the embedded Pt NCs could serve as the nucleus to regulate the nucleation and growth kinetics of the Fe_2_O_3_ growth, resulting in the denser and smaller morphology. Because of the worm‐like structure becomes much denser and smaller, increasing the film surface roughness causes contact angle dropping from 38.0° to 27.1° (Figure S4, Supporting Information), resulting in a more hydrophilia Fe_2_O_3_@Pt surface, which is conducive to the full contact between the electrolyte and the reactive site on the film surface. XRD patterns and Raman spectroscopy (Figure S5, Supporting Information) showed that the crystal structure of Fe_2_O_3_@Pt was not affected by the incorporation of Pt NCs, and no new peak and obvious peak shift were found. To explore the position relationship between Pt NCs and Fe_2_O_3_ film, TEM characterization was performed. Figure 2g,h shows the TEM and HRTEM of Fe_2_O_3_@Pt film. Figure 2g reveals that the existence of Pt NCs, and two lattice fringes can be seen from HRTEM (Figure 2h), corresponding to the (111) crystal plane of Pt and the (112) crystal plane of α‐Fe_2_O_3_, respectively.

### 2.3 PEC Water Splitting Performance of Fe_2_O_3_@Pt

**Figure 3:**
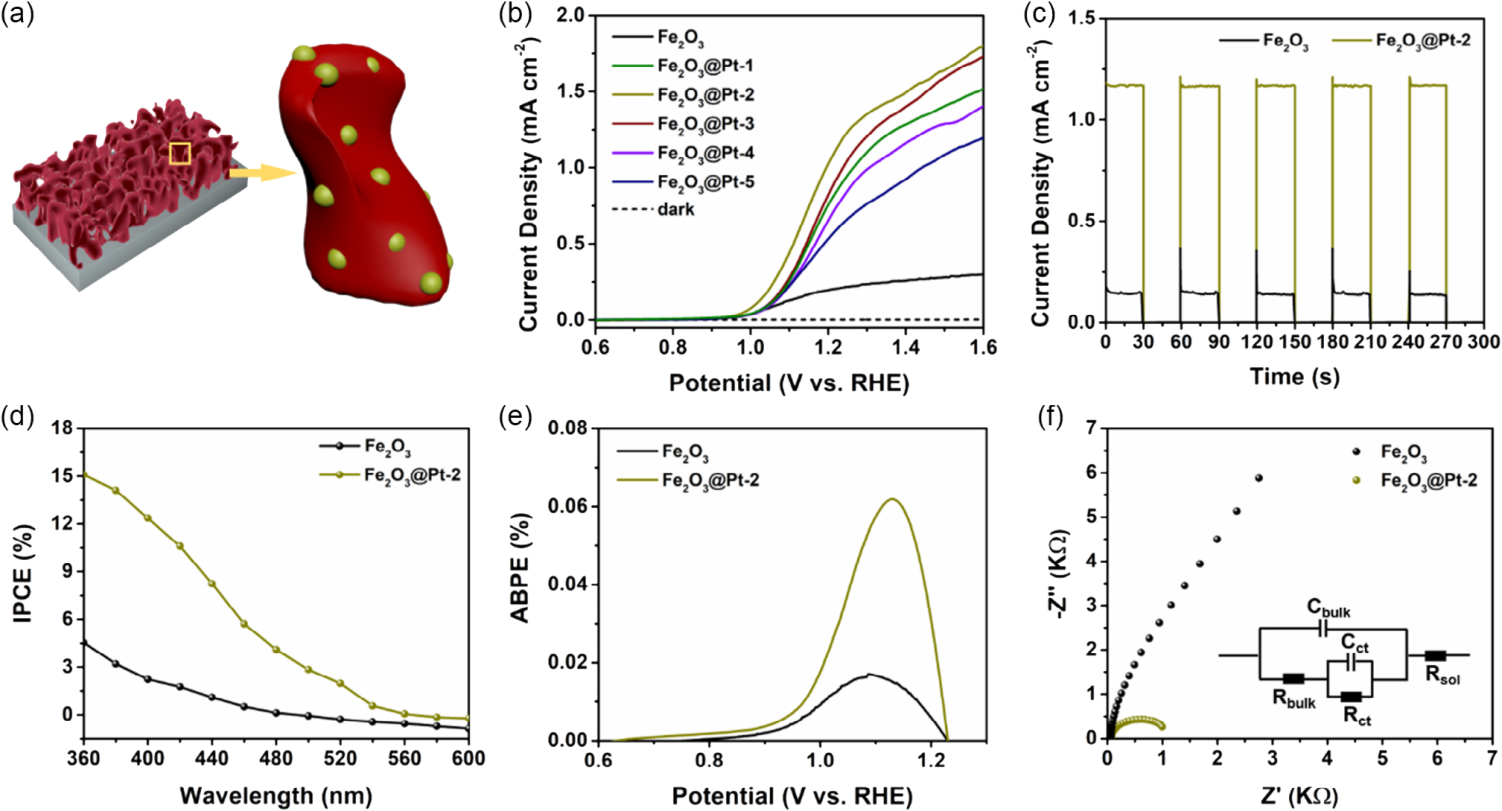
a) Schematic illustration of Fe_2_O_3_@Pt films. b) *J–V* curve of Fe_2_O_3_ and different Fe_2_O_3_@Pt films. c) Transient photocurrent measurements of Fe_2_O_3_ and Fe_2_O_3_@Pt‐2 films. d) IPCE spectra. e) ABPE spectra. f) Electrochemical impedance spectra (EIS) curves under irradiation of Fe_2_O_3_ and Fe_2_O_3_@Pt‐2 film.

Next, we test the photoelectrochemical performance of Fe_2_O_3_@Pt photoelectrode (**Figure** **3**a) under AM 1.5G illumination (100 mW cm^−2^) to evaluate how the embedding of Pt NCs impacts the PEC water splitting performance of Fe_2_O_3_ films. Figure 3b shows the linear voltammetric sweep curves of the Fe_2_O_3_ films with different concentrations of Pt NCs. AM 1.5G simulated sunlight irradiates from the back of the film. It can be seen from the figure that the photocurrent density of the film without Pt NCs is 0.21 mA cm^−2^ at 1.23 V_RHE_. With the increase of the concentration of Pt NCs, the performance of the sample first increases and then decreases. Fe_2_O_3_@Pt‐2 has the highest photocurrent density of 1.16 mA cm^−2^ at 1.23 V_RHE_. Further, the transient photocurrent measurements of pristine Fe_2_O_3_ and Fe_2_O_3_@Pt‐2 films were performed at 1.23 V_RHE_ under chopped light illumination (Figure 3c) to assess the charge recombination behavior at the semiconductor–electrolyte junction. Compared with the Fe_2_O_3_ film, Fe_2_O_3_@Pt‐2 films can achieve the steady state photocurrent faster, manifesting the significantly improved charge separation efficiency of photoelectrode caused by the embedding of Pt NCs.

Figure S6, Supporting Information, shows the UV–vis absorption curves of Fe_2_O_3_ and Fe_2_O_3_@Pt‐2 films. It can be seen from the figure that the absorption limits of the two films are about 600 nm, In the range of 360–480 nm, two samples show certain optical absorption characteristics, and the difference is not big. Figure 3d and S7, Supporting Information, show the incident photon‐to‐current conversion efficiency (IPCE) curves of the Fe_2_O_3_ films with different concentrations of Pt NCs. It is obvious from the figure that the IPCE values of all Fe_2_O_3_ films are very low (less than 3%) in the wavelength range of 520–600 nm, and the IPCE values increase rapidly with the decrease of wavelength in the wavelength range of 520–360 nm. The champion efficiency of Fe_2_O_3_@Pt‐2 film could reach up to 15.1%, while the IPCE of the Fe_2_O_3_ is lower than 5% through the entire responsive region. Figure 3e shows the applied bias photon‐to‐current efficiency (ABPE) curves of Fe_2_O_3_ and Fe_2_O_3_@Pt‐2 films. ABPE reflects the photoelectric conversion efficiency of the sample under applied bias. It can be seen from the figure that the maximum ABPE value of Fe_2_O_3_ film without Pt NCs is 0.017%, and the maximum ABPE value of Fe_2_O_3_@Pt‐2 could reach up to 0.062%, the IPCE and ABPE results are summarized in Table S2, Supporting Information. Therefore, the implantation of Pt NCs can improve the photoelectric conversion efficiency of the films, and further improve their photoelectrochemical properties.

To further characterize the effect of Pt NCs implantation on the properties of the samples, electrochemical impedance spectroscopy (EIS) and Mott–Schottky (MS) tests were carried out. Figure 3f shows the electrochemical impedance spectra of Fe_2_O_3_ and Fe_2_O_3_@Pt‐2 films. Table S3, Supporting Information, shows the solution resistance *R*_sol_, the transfer resistance *R*_bulk_ inside the film, and the charge transfer resistance *R*_ct_ of Fe_2_O_3_ and Fe_2_O_3_@Pt‐2 films.^[^ ^43^ ^]^ It can be clearly seen that the solution resistance *R*_sol_ of two films has no obvious change, while the interfacial properties (charge transfer resistance *R*_ct_) change greatly. The charge transfer resistance *R*_ct_ of Fe_2_O_3_ films without Pt NCs is 8425 Ω, while *R*_ct_ of Fe_2_O_3_@Pt‐2 is 619 Ω, which indicates the decrease of surface recombination after embedding Pt NCs. Meanwhile, the significant decrease of *R*_bulk_ suggests that more efficient carrier separation and transport inside Fe_2_O_3_@Pt‐2 film.^[^ ^44^ ^]^ Combined with the SEM image, the decrease of charge transfer resistance of Fe_2_O_3_@Pt‐2 may be due to the worm‐like structure becoming much denser and smaller, good contact between particles, and promoted charge transfers due to the addition of Pt NCs. MS curves (Figure S8, Supporting Information) reveal that the implantation of Pt NCs does not change the type of Fe_2_O_3_ (n‐type), but results in much shallower slopes, indicating higher carrier densities.^[^ ^37^ ^]^ And the increase of carrier densities will shift the Fermi level of the bulk (measured potential) toward more cathodic potentials for Fe_2_O_3_@Pt films.^[^ ^45^ ^]^ Therefore, the addition of Pt NCs can reduce the charge transfer resistance of Fe_2_O_3_ thin films, increase the concentration of carriers, which is conducive to carrier transport, and improve the photoelectrochemical properties of Fe_2_O_3_ thin films.

It can be found from the SEM images and contact angle results that the implantation of Pt NCs affects the size of worm‐like particles and the surface state of the samples. Figure S9, Supporting Information, shows the cyclic voltammetry curves and estimated double‐layer capacitance of Fe_2_O_3_ and Fe_2_O_3_@Pt‐2 films at different scanning rates. As shown in Figure S9c, Supporting Information, the electrochemical active surface area (ECSA) value of Fe_2_O_3_@Pt‐2 film is 5.1 μF cm^−2^, which is higher than that of Fe_2_O_3_ film (3.9 μF cm^−2^). Therefore, the implantation of Pt NCs is beneficial to improve the ECSA of the samples, provide more active sites in the oxygen evolution reaction, and improve the photoelectrochemical properties.

**Figure 4:**
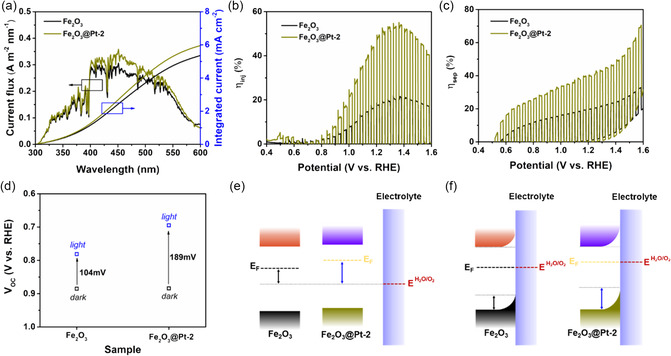
a) The calculated current density flux and integrated current density (*J*_abs_) of Fe_2_O_3_ and Fe_2_O_3_@ Pt‐2 films. b) Surface charge injection efficiency (*η*_inj_) of Fe_2_O_3_ and Fe_2_O_3_@ Pt‐2 films. c) Bulk charge separation efficiency (*η*_sep_) of Fe_2_O_3_ and Fe_2_O_3_@ Pt‐2 films. d) Open‐circuit potential of Fe_2_O_3_ and Fe_2_O_3_@Pt‐2 films. Schematic diagram of band structures of Fe_2_O_3_ and Fe_2_O_3_@Pt‐2 films: e) before and f) after contacting with the electrolytes.

To analyze the effect of Pt nanocrystals implantation on carrier transfer, we measured the surface charge injection efficiency (*η*_inj_) and the bulk charge separation efficiency (*η*_sep_) of Fe_2_O_3_ and Fe_2_O_3_@Pt‐2 films. The values of *η*_inj_ and *η*_sep_ were based on the calculation according to Equation (S6) and (S7), Supporting Information, where $J_{H_{2}O}$, $J_{H_{2}O_{2}}$, light harvesting efficiency, and *J*_abs_ for each photoanode are shown in **Figure** **4**a and S10, Supporting Information, and these values are summarized in Table S4, Supporting Information. It can be seen that the *η*_inj_ of the Fe_2_O_3_@Pt‐2 film increases (Figure 4b), at 1.23 V_RHE_, *η*_inj_ increased from 19.4% (Fe_2_O_3_) to 48.3% (Fe_2_O_3_@Pt‐2). Furthermore, the above tendency was also observed for *η*_sep_ at 1.23 V_RHE_ for the Fe_2_O_3_@Pt‐2 with respect to Fe_2_O_3_ (Figure 4c). At 1.23 V_RHE_, *η*_sep_ increased from 20.1% to 40.1%. The above *η*_inj_ and *η*_sep_ results indicate that the implantation of Pt NCs can promote the separation of bulk carriers and accelerate the more holes transfer to photoelectrode/electrolyte interface, resulting in the more efficient PEC water oxidation reaction.

Open circuit voltage (OCP) is the difference between the quasi‐Fermi level of the electron–hole. The difference between the OCP under the condition of on light and off light is called photovoltage, which is the driving force to inject the photogenerated hole into the electrolyte. Figure 4d and S11, Supporting Information, show the OCP of Fe_2_O_3_ films with different concentrations of Pt NCs. After embedding the Pt NCs, the photovoltage of the films increased significantly, which indicates that the driving force of electron–hole separation is enhanced after adding Pt NCs. The photovoltage of the Fe_2_O_3_ film is 104 mV, while that of the Fe_2_O_3_@Pt‐2 film is 189 mV, and the OCPs are repeatable under intermittent irradiation (Figure S11b and Table S5, Supporting Information). This indicates that the driving force of electron–hole separation is enhanced after adding Pt NCs.

### 2.4 Unravelling the Roles of Pt Nanocrystals of Fe_2_O_3_ Photoanode

The efficient charge transfer inside Fe_2_O_3_@Pt‐2 film was further confirmed by intensity modulated photo‐current spectroscopy (IMPS). Figure S12a,b, Supporting Information, shows typical IMPS responses of Fe_2_O_3_ and Fe_2_O_3_@Pt‐2 films. And according to Equation (S10) and (S11), Supporting Information, we can extract the values of carrier transport time *τ*_d_, charge recombination constants *k*_rec_, transport rate constants *k*_trans_, and charge transfer efficiency (*k*_tran_/(*k*_tran_ + *k*_rec_)), and these values are summarized in Table S6, Supporting Information. As shown in Figure S12c, Supporting Information, the value of *k*_trans_ is 111.8 s^−1^ for Fe_2_O_3_@Pt‐2 film, which is about 12 times larger than that of Fe_2_O_3_ (9.09 s^−1^), while the carrier transport time *τ*_d_ is lesser (0.05 ms) for Fe_2_O_3_@Pt‐2 than that for Fe_2_O_3_ (0.184 ms), which is in good agreement with the EIS and OCP measurement. Meanwhile, the charge transfer efficiency (*k*_tran_/(*k*_tran_ + *k*_rec_)) can be improved from 0.17 (Fe_2_O_3_) to 0.87 (Fe_2_O_3_@Pt‐2) (Figure S12d, Supporting Information). These results indicate that the implantation of Pt NCs in during photocatalysis process, not only is prone to speed up the carrier transfer inside photoelectrode, but also reduces charge recombination.^[^ ^46^, ^47^ ^]^

To explain this phenomenon, we build a model of Fe_2_O_3_@Pt, as shown in Figure 3a, Pt NCs were uniformly embedded in Fe_2_O_3_ particles. Because the work function of Pt is larger than that of Fe_2_O_3_, the Schottky barrier can be formed when Pt and Fe_2_O_3_ contact (Figure S13a,b, Supporting Information).^[^ ^45^ ^]^ At this time, photogenerated electrons can be transferred from Fe_2_O_3_ to Pt, which can reduce the recombination of electrons and holes. Different from the Schottky barrier formed by two semiconductors, the electrons transferred from Fe_2_O_3_ to Pt metal will not accumulate, and usually drift current is formed to transfer away, which is because Pt metal has excellent conductivity. In addition, the formed build‐in electric field between Fe_2_O_3_ and Pt, as shown schematically in Figure S13c, Supporting Information, which provides an extra channel to facilitate the photogenerated holes transfer and collection. Therefore, the implantation of Pt nanocrystals can not only promote the separation of photogenerated electrons and holes, but also transfer the photogenerated holes quickly, which improves the carrier transport dynamics and improves the photoelectrochemical properties of the films.

Furthermore, the ultraviolet–visible diffuse reflectance spectra (UV–vis DRS) and ultraviolet photoelectron spectroscopy (UPS) analysis of Fe_2_O_3_ and Fe_2_O_3_@Pt‐2 films are shown in Figure S14, Supporting Information. The UV–vis DRS in Figure S14a, Supporting Information, revealed that the optical bandgap of the Fe_2_O_3_ and Fe_2_O_3_@Pt‐2 samples is 2.11 and 2.10 eV, respectively. The much narrower bandgap of Fe_2_O_3_@Pt‐2 will increase carrier densities, resulting the highest PEC performance of Fe_2_O_3_@Pt‐2. The positions of the valence band maximum (VBM) and conduction band minimum (CBM) were estimated to be 2.05 and –0.06 V_RHE_ for Fe_2_O_3_, and 1.99 and –0.11 V_RHE_ for Fe_2_O_3_@Pt‐2 by UPS spectra in Figure S14b,c, Supporting Information, which is calculated on the previously published.^[^ ^48^ ^]^ Using VBM, CBM, Fermi levels, and bandgap, as shown in Figure 4e,f and Table S7, Supporting Information, we constructed band diagrams of Fe_2_O_3_ and Fe_2_O_3_@Pt‐2 films before and after contacting with the electrolytes. It can be seen that the implantation of Pt NCs has a positive effect on the band structure of Fe_2_O_3_ films, the lower Fermi level of Fe_2_O_3_@Pt‐2 is resulted from the increase of carrier densities.^[^ ^45^ ^]^ Fe_2_O_3_@Pt‐2 has lower Fermi level than that of Fe_2_O_3_, showing the largest initial energy difference (Fermi level difference between Fe_2_O_3_ and electrolyte), resulting the widest depletion width to efficiently separate electron–hole pairs.^[^ ^48^, ^49^ ^]^

## 3 Discussion

**Figure 5:**
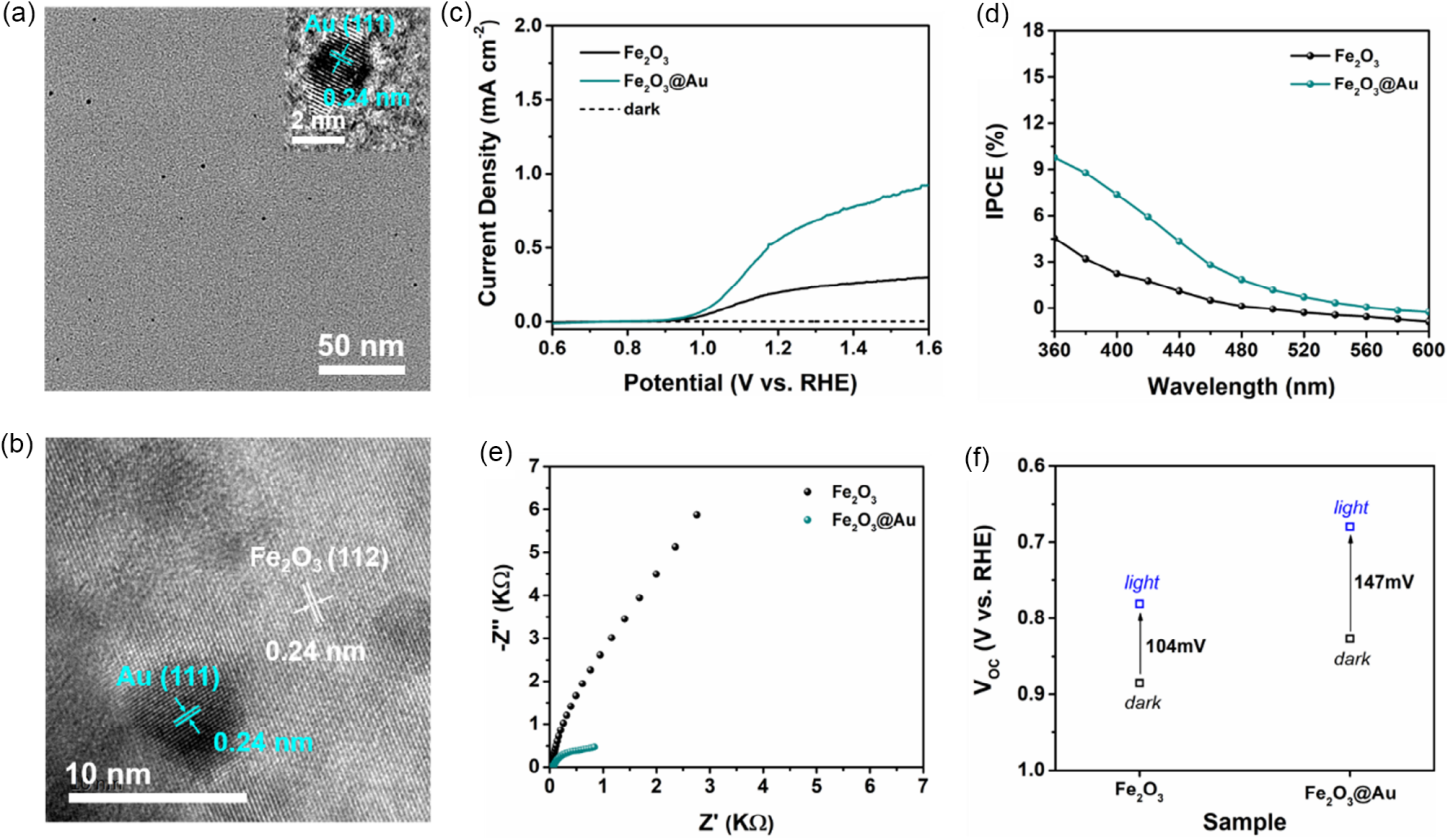
a) TEM image of Au nanocrystals (insert: HRTEM image). b) HRTEM images of Fe_2_O_3_@Au films. c) *J–V* curve of Fe_2_O_3_ and Fe_2_O_3_@Au films. d) IPCE spectra. e) EIS curves under irradiation of Fe_2_O_3_ and Fe_2_O_3_@Au film. f) Open‐circuit potential of Fe_2_O_3_ and Fe_2_O_3_@Au films.

We further prepared sub‐5 nm ligand‐free Au nanocrystals (Au NCs) in ethanol (precursor solvents) through the PLIL (**Figure** **5**a and S15a, Supporting Information), and then introduced Au NCs into the Fe_2_O_3_ film via spin‐coating method (Figure 5b and S15d, Supporting Information). The prepared Fe_2_O_3_@Au films show worm‐like nanoporous structure, as shown in Figure S15b,c, Supporting Information, the worm‐like structure becomes much denser and smaller than Fe_2_O_3_ (Figure 2a and 4c). The crystal structure of Fe_2_O_3_@Au film shows no obvious change from XRD and Raman results after the implantation of Au NCs (Figure S16, Supporting Information). The photocurrent density of Fe_2_O_3_@Au could enhance up to 0.62 mA cm^−2^ at 1.23 V_RHE_, and the IPCE and ABPE spectra of Fe_2_O_3_@Au film also show obvious enhancement compared with that of Fe_2_O_3_ films (Figure 4d and S17, Supporting Information). And the above increase tendency was also observed for the surface charge injection efficiency (*η*_inj_) and the bulk charge separation efficiency (*η*_sep_) of Fe_2_O_3_@Au (Figure S18, Supporting Information), indicating that the implantation of Au NCs also could promote the separation of bulk carriers and more holes can arrive at the photoelectrode/electrolyte interface. Further, the EIS, MS, IMPS, OCP, and ECSA results of Fe_2_O_3_@Au film also demonstrate that the implantation of Au NCs could accelerate the carrier separation and transfer (Figure 5f, S19–S22, and Table S2–S6, Supporting Information), which is consistent with the results of the implantation of Pt NCs.

Remarkably, the photoelectrochemical performance of Fe_2_O_3_@Au photoanode is lower than Fe_2_O_3_@Pt photoanode (Table S2–S6, Supporting Information), which is related to the initial energy difference. As shown in Figure S23 and S24, Supporting Information, the trend of the initial energy difference as follows: Fe_2_O_3_@Pt‐2 > Fe_2_O_3_@Au > Fe_2_O_3_, resulting in the trend of driving force for carrier transfer as follows: Fe_2_O_3_@Pt‐2 > Fe_2_O_3_@Au > Fe_2_O_3_, thus the Fe_2_O_3_@Pt‐2 photoanode exhibited the champion photoelectrochemical performance.

## 4 Conclusion

In summary, this work demonstrates an efficient strategy for embedding laser‐generated Pt nanocrystals to improve the Fe_2_O_3_ photoanode PEC performance. Compared with the traditional solution method, the advantage of liquid phase pulsed laser irradiation technology is that the range of solvent is wide, and the nanocrystals can be prepared in situ in the solvent, and the obtained nanocrystals often do not contain ligands, which is conducive to the transmission of electrons. Different from Pt element doping, the implantation of Pt nanocrystals could construct local nano‐heterointerfaces in Fe_2_O_3_@Pt film, which could provide an extra channel to promote the carrier separation and facilitate the photogenerated holes transfer and collection. The EIS of the Fe_2_O_3_ film modified by Pt nanocrystals decreases and the carrier separation efficiency improves, leading to significantly increased photocurrent density, i.e., 1.16 mA cm^−2^ at 1.23 V_RHE_ (1M KOH). This work provides an alternative way to improve the PEC performance of Fe_2_O_3_.

## Conflict of Interest

The authors declare no conflict of interest.

## Supporting information

Supplementary Material

## Data Availability

The data that support the findings of this study are available in the supplementary material of this article.
