# Supplementary material for: Boosting the Solar Water Oxidation Performance of Fe2O3 Photoanode via Embedding Laser‐Generated Pt Nanocrystals
Source: Small Sci. 2024 Feb 27;4(5):2300318. doi: 10.1002/smsc.202300318 (PMC11935253; doi:10.1002/smsc.202300318)
Supplement: Supplementary file 1 — Supplementary Material [file SMSC-4-2300318-s001.pdf]

Supporting Information

Boosting the Solar Water Oxidation Performance of Fe_2O_3 Photoanode via Embedding Laser Generated Pt Nanocrystals

Fan Li, Jie Jian, Shiyuan Wang, Lichao Jia, and Hongqiang Wang**

Experimental section

Materials

Fluorine-doped tin oxide (FTO) substrates (~14 Ω sq^{-1}) were obtained from Pilkington. Block copolymer (F-127) and Iron nitrate ($Fe(NO_3)_3{\cdot}9H_2O$, 98+%) were obtained from Sigma-Aldrich Co. LLC. Pt metal sheet was obtained from Zhongnuo Advanced Material. Potassium hydroxide (KOH), acetone (C_3H_6O, 99.5%), and ethanol (C_2H_5OH, 99.5%) were obtained from Sinopharm Chemical Reagent Co., Ltd. All materials were used as received without any further purification. And FTO substrates were ultrasonically pre-cleaned before sample preparation in ultrapure water, ethanol, and acetone, then dried by N_2 flow and treated by an ultraviolet ozone cleaner (SC-UV-I, SETCAS Electronics Co., Ltd).

Laser generated Pt nanocrystals

Pt metal sheet was firstly immersed in 10 mL ethanol. And then, at room temperature, above the mixture was illuminated for 5 minutes by 1064 nm unfocused laser beam with a laser fluence of 1.0 J $pulse^{-1}$ cm^{-2}, Pt nanocrystals colloidal solution can be obtained, and the concentration of Pt NCs is around 0.28 mg mL^{-1}.

Preparation of the Fe_2O_3 photoanodes

Fe_2O_3 photoanodes were fabricated by spin-coating deposition. By using 40 °C water bath dissolving 0.26 g F127 in 2.0 mL ethanol with stirring for 1h (solution I). And at room temperature, dissolve 0.08 g Fe $(NO_3)_3{\cdot}9H_2O$ in another 2.0 mL ethanol with stirring for 1h (solution II). Then, the precursor solution is obtained by mixing solution I and solution II were together and stirring for 2h at room temperature. Finally, Fe_2O_3 photoanodes were fabricated by spin-coating deposition. The precursor solution was deposited on cleaned FTO substrates with 2000 rpm for 20 seconds and 4000 rpm for 40 seconds followed by baking at 350 °C for 10 min, these procedures were repeated thrice. Subsequently, above samples were annealed at 500 °C for 2 hours and 800°C for 10 min, the Fe_2O_3 photoanodes can be obtained.

Embedding Pt nanocrystals in Fe_2O_3 photoanodes

Firstly, similar to the preparation of Fe_2O_3 photoanodes, solution I and solution II are obtained and then mixed together. Subsequently, 0.2 mL Pt nanocrystals colloidal solution and 0.8 mL ethanol were added to the above mixed solution with stirring for 1h, the precursor solution can be obtained. After the procedures of spin-coating deposition and annealing, the Fe_2O_3@Pt-1 (0.056 mg Pt nanocrystals) film is obtained. Further, Fe_2O_3@Pt-2 (0.4 mL Pt nanocrystals colloidal solution and 0.6 mL ethanol, 0.112 mg Pt nanocrystals), Fe_2O_3@Pt-3 (0.6 mL Pt nanocrystals colloidal solution and 0.4 mL ethanol, 0.168 mg Pt nanocrystals), Fe_2O_3@Pt-4 (0.8 mL Pt nanocrystals colloidal solution and 0.2 mL ethanol, 0.224 mg Pt nanocrystals), Fe_2O_3@Pt-5 (1.0 mL Pt nanocrystals colloidal solution and 0.0 mL ethanol, 0.280 mg Pt nanocrystals), were obtained by adding different volumes of Pt nanocrystals colloidal solution.

Characterization

The XRD spectra were investigated by the Panalytical (X'pert PRO) for the crystal structures of the as-synthesized samples. The Raman spectra were investigated by the Renishaw inVia RM200 (Renishaw) with a 532 nm laser. XPS spectra were measured by the Axis Supra (Kratos). The surface morphology and microstructure of the as-synthesized samples were analyzed using the field emission scanning electron microscope (SEM, FEINovaNanoSEM 450) and the high-resolution transmission electron microscopy (HRTEM, Talos F200X).

Photoelectrochemical measurements

The photoelectrochemical measurements of the as-synthesized photoanodes were investigated at room temperature by the three-electrode cell (working electrodes: photoanodes; counter electrodes: Pt wire; reference electrodes: Ag/AgCl electrode, saturated KCl) and an electrochemical workstation (CHI660E). The light source (AM 1.5G filter, 100 mW cm^{-2}) is Xe 500 W lamp (CEL-S500). The working area of photoanode was 0.25 cm^2. Noticeably, the J_{H2O2} the as-synthesized photoanodes are obtained in 1 M KOH with 0.1 M H_2O_2 (hole trapping agent) electrolyte. In addition, the electrolyte is 1 M KOH standard aqueous solution for all the other photoelectrochemical measurements of the as-synthesized photoanodes.

J-V curves were obtained under the irradiation in the anodic direction with the potential range from -1.1 to 0.4 V vs. Ag/AgCl with a scan rate of 10 mV s^{-1}. EIS spectra were obtained in 1 M KOH electrolyte (frequency range: 0.1 Hz - 1MHz) at 1.23 V_{RHE} under illumination. Intensity-modulated photocurrent spectroscopy (IMPS) data were obtained at 1.23 V_{RHE} in 1 M KOH electrolyte (frequency range: 0.1 Hz – 100 kHz). Cold white LED is used as the light source (420nm, AM 1.5G spectrum), The modulated light intensity is set to 10% of the background light intensity. The PEC measurements of all photoanodes were performed under AM1.5 illumination from the back (FTO substrate side).

The recorded potentials versus Ag/AgCl ($E_{Ag/AgCl}$) were converted against reversible hydrogen electrode (RHE) using the Nernst equation:[S1]

$$E_{RHE} = E_{Ag/AgCl} + 0.1976 + 0.0591 \times pH \qquad \text{Equation 1}$$

where E_{RHE} is the converted potential referred to the RHE and $E_{Ag/AgCl}$ is the experimentally measured potential against the Ag/AgCl reference electrode.

The incident photon-to-current conversion efficiency (IPCE) was confirmed using a monochromator (CEL-IS151) with a 300 W Xe light (CEL-HXF300) source, according to the equation:[S2]

$$IPCE = \frac{1240 J_{photo}}{\lambda P} \qquad \text{Equation 2}$$

where λ is the wavelength, J_{photo} is the photocurrent density under monochromatic light, and P is the measured irradiance under monochromatic light.

The absorbed photon-to-current efficiency (APCE) is calculated based on the following equation:[S1]

$$APCE = \frac{IPCE}{LHE} \qquad \text{Equation 3}$$

The light absorption efficiency or light harvesting efficiency (LHE, defined as the ratio of absorbed light to the incident light) of each photoanode is calculated from the UV–vis absorption spectra:

$$LHE = 1 - 10^{-A(\lambda)} \qquad \text{Equation 4}$$

where A(λ) is the absorbance at wavelength λ.

The applied bias photon-to-current efficiency (ABPE) was also used to quantify the photoanode

performance following the below equation:[S3]

$$ABPE = \frac{J_{photo}(1.23 - V_{app})}{P_{light}} \quad \text{Equation 5}$$

in which J_{photo} is the photocurrent density, V_{app} is the applied bias (V vs. RHE), and P_{light} is the incident light density (100 mW cm^{-2}).

The efficiency of charge transport in the surface (η_{inj}) and bulk (η_{sep}) for the prepared photoanodes can be calculated using the following equations:[S4]

$$\eta_{inj} = \frac{J_{H_2O}}{J_{H_2O_2}} \quad \text{Equation 6}$$

$$\eta_{sep} = \frac{J_{H_2O_2}}{J_{abs}} \quad \text{Equation 7}$$

J_{abs} is the unity converted photocurrent density from the light absorption, while J_{H2O} and J_{H2O2} are the photocurrent densities obtained in 1 M KOH electrolyte and 1 M KOH with 0.1 M H_2O_2, respectively.

J_{abs} can be calculated according to the following equation:[S3]

$$J_{abs} = \int N_{ph}(\lambda) \times LHE(\lambda) \times e d\lambda \quad \text{Equation 8}$$

where $N_{ph}(\lambda)$ is the photon flux (mW cm^{-2} nm^{-1}), e is the electronic charge (1.602×10^{-19} C).

Electrochemical impedance spectra (EIS) measurements were carried out at frequency range of 1 MHz to 10 Hz by applying at 1.23 V_{RHE} under the illumination. M-S plots were measured at a frequency of 1000 Hz in 1 M KOH aqueous solution under dark condition. According to the M-S curve, charge carrier density (Nd) can be calculated using the following equation:[S5]

$$N_d = \frac{2}{e\varepsilon_0\varepsilon} \times \left[\frac{d\left[\frac{1}{C^2}\right]}{dV}\right]^{-1} \quad \text{Equation 9}$$

The ε_0 is vacuum permittivity (8.85×10^{-12} C^2 J^{-1} m^{-1}), and ε is relative permittivity (32 for hematite).[S6]

The electrochemical active surface area (ECSA) was estimated from the electrochemical double-layer capacitance according to a previous published report.[S7, S8] Cyclic voltammograms were performed in 1 M KOH (pH = 13.6) at the scan rate of 20, 40, 60, 80, and 100 mV s^{-1}. Then the electrochemical active surface area was determined by measuring the capacitive current associated with double-layer

charging from the scan-rate dependence of CVs. The double layer capacitance (C_{dl}) was estimated by plotting the ΔJ at 0 V_{RHE} against the scan rate. The linear slope is equivalent to twice of the C_{dl}, which can be used to represent the electrochemical active surface area.

Carriers transport time (τ_d):[S9]

$$\tau_d = (2\pi f_{IMPS})^{-1} \qquad \text{Equation 10}$$

in which, f_{IMPS} is the minimum frequency in IMPS spectra.

The low frequency intercept represents the charge transfer efficiency (k_{tran}/ (k_{tran} + k_{rec})):[S10, S11]

$$\omega_{max} =(2\pi f_{max})^{-1} = k_{tran} + k_{rec} \qquad \text{Equation 11}$$

As outlined above, we can obtain the relative rate constants k_{rec} and k_{tran}.

Open circuit photovoltage (OCP) was carried out under illumination (OCV_{light}) and dark (OCV_{dark}) in 1 M KOH, respectively:

$$OCP = OCV_{light} - OCV_{dark} \qquad \text{Equation 12}$$

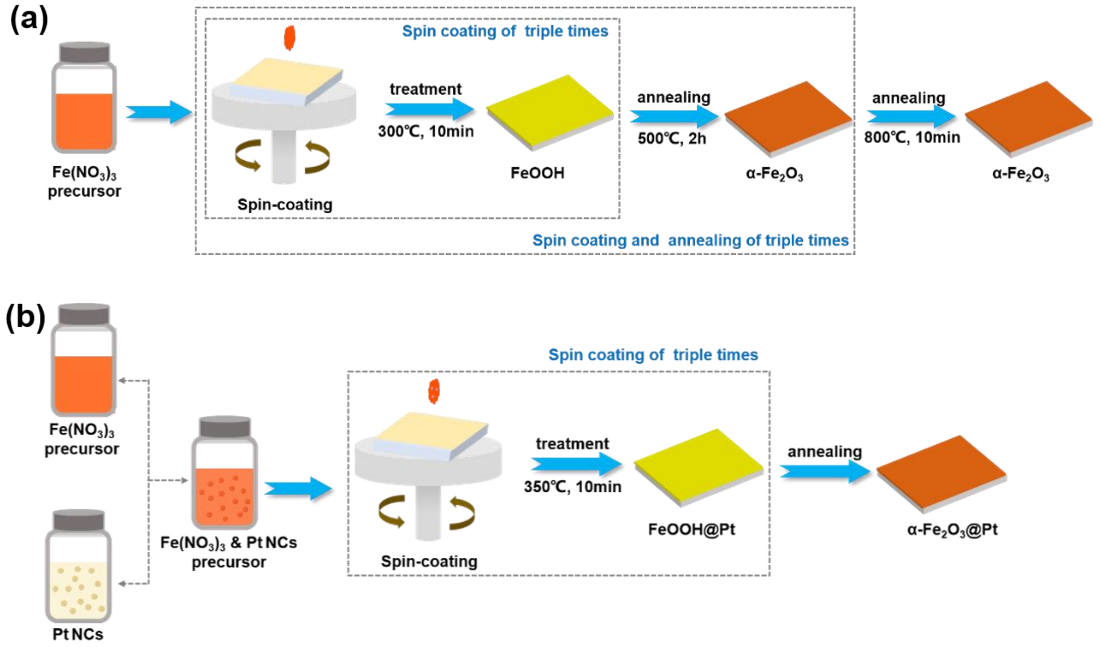

Figure S1. Schematic illustration of the fabrication procedure of (a) Fe_2O_3 and (b) Fe_2O_3@Pt films by spin-coating.

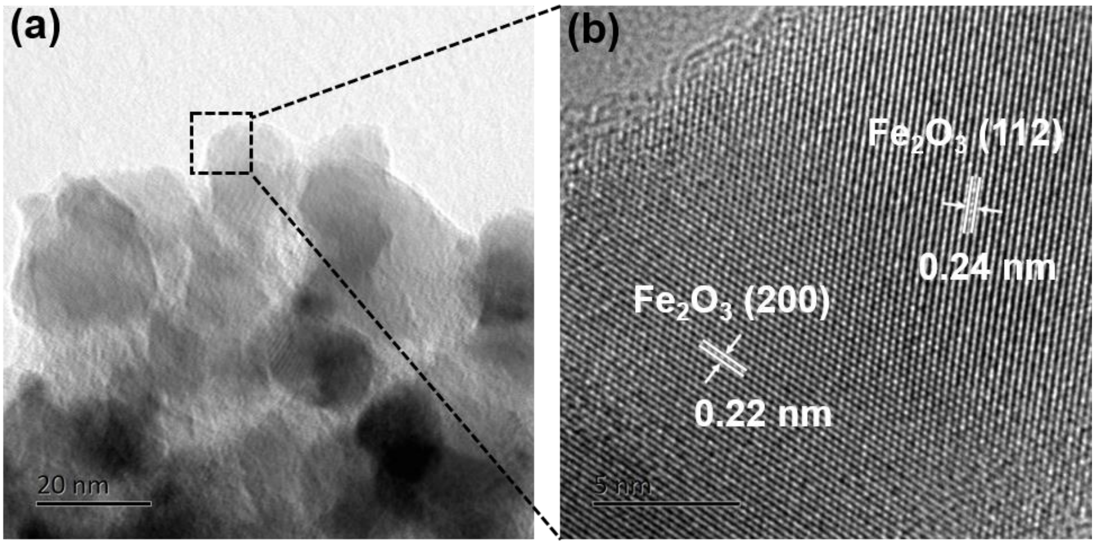

Figure S2. (a) TEM images and (b) HRTEM images of Fe_2O_3 films.

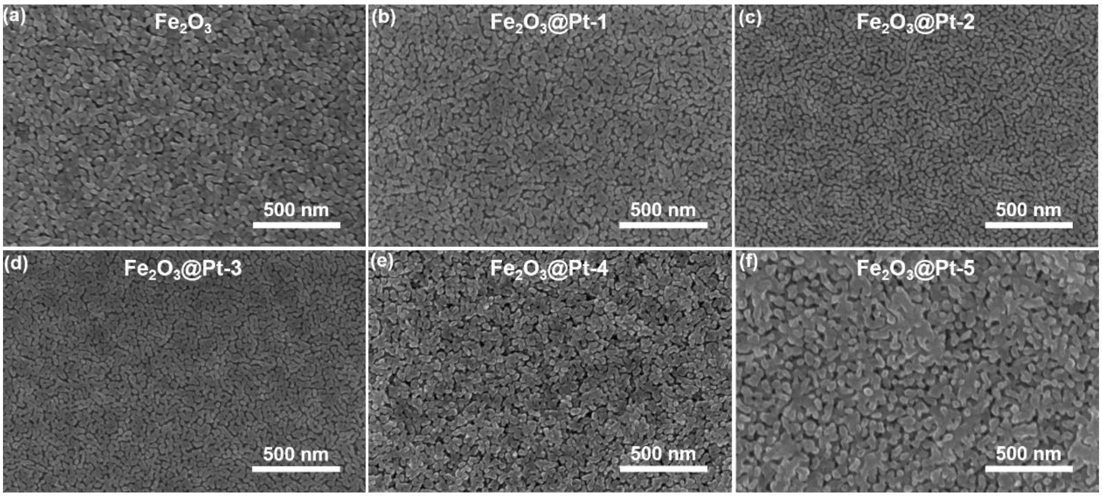

Figure S3. Surface SEM images of (a) Fe_2O_3, (b) Fe_2O_3@Pt-1, (c) Fe_2O_3@Pt-2, (d) Fe_2O_3@Pt- 3, (e) Fe_2O_3@Pt- 4, (f) Fe_2O_3@Pt-5 films.

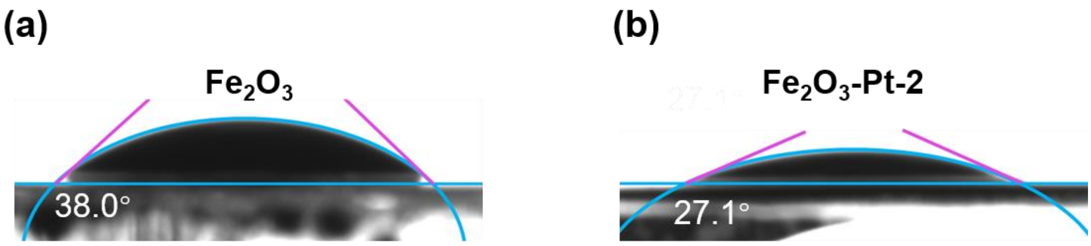

Figure S4. Contact angles of water on the (a) Fe_2O_3, and (b) Fe_2O_3@Pt-2 films

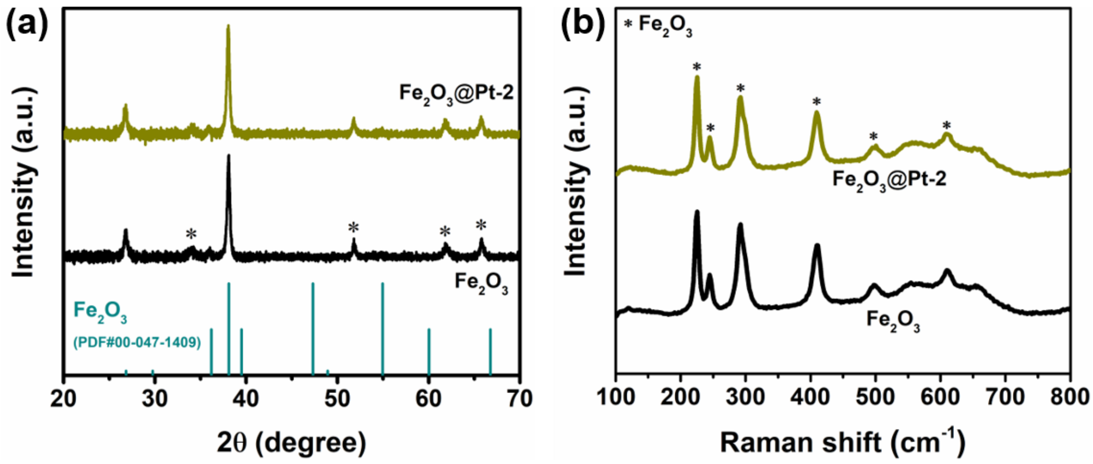

Figure S5. (a) XRD pattern, and (b) Raman spectra of Fe_2O_3 and Fe_2O_3@Pt-2 films.

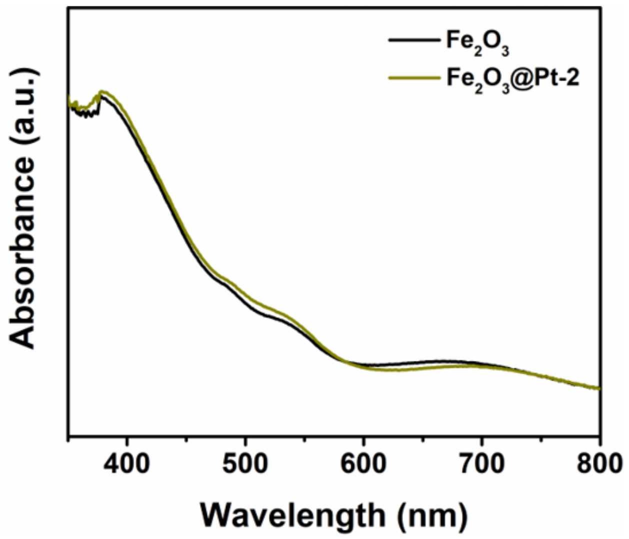

Figure S6 UV-Vis spectra of Fe_2O_3 and Fe_2O_3@Pt-2 films.

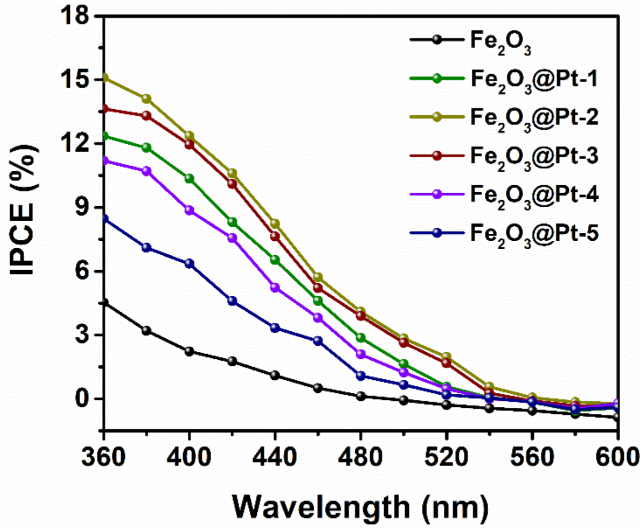

Figure S7 IPCE curves of the Fe_2O_3 films with different concentrations of Pt NCs.

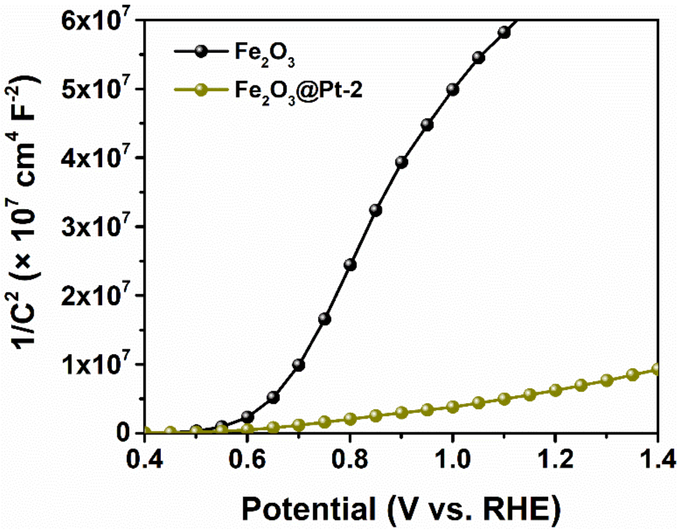

Figure S8 Mott-Schottky (M-S) plots measured in 1M KOH solution at 1 kHz frequency of Fe_2O_3 and Fe_2O_3@ Pt-2 films.

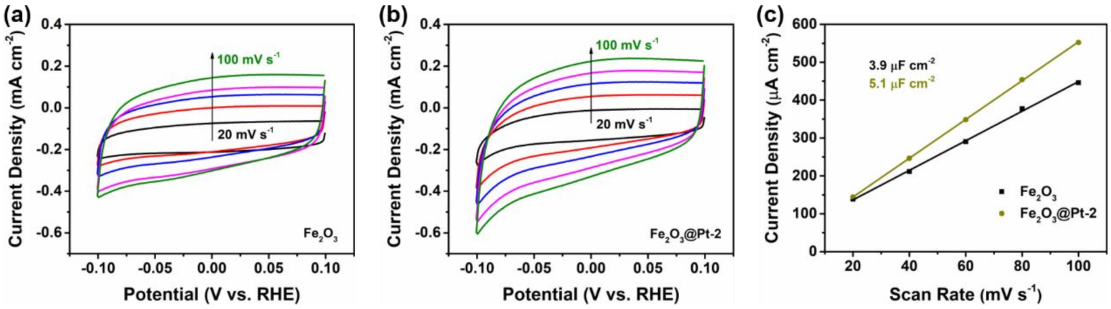

Figure S9 Typical cyclic voltammetry curves in 1M KOH of (a) Fe_2O_3 and (b) Fe_2O_3@Pt-2 films. (c) Estimate of the electrochemical active surface area of Fe_2O_3 and Fe_2O_3@Pt-2 films

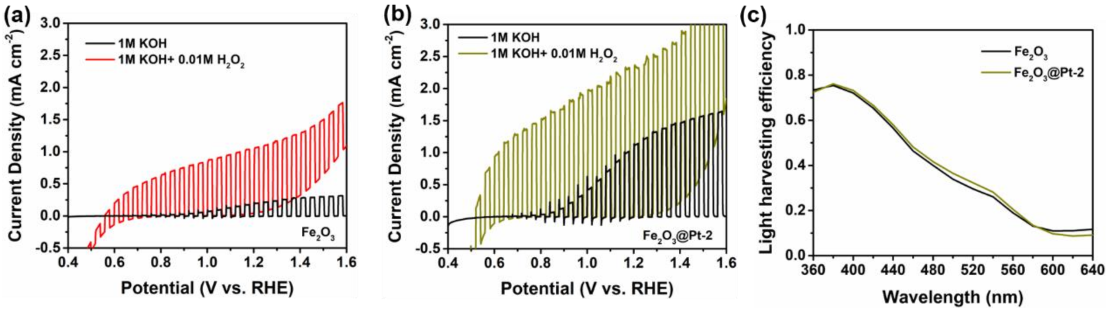

Figure S10 *J-V* curve of (a) Fe_2O_3 and (b) Fe_2O_3@ Pt-2 in 1M KOH solution with 0.1 M H_2O_2 under AM 1.5G illumination (100 mW cm^{-2}). (c) LHE of Fe_2O_3 and Fe_2O_3@ Pt-2 films.

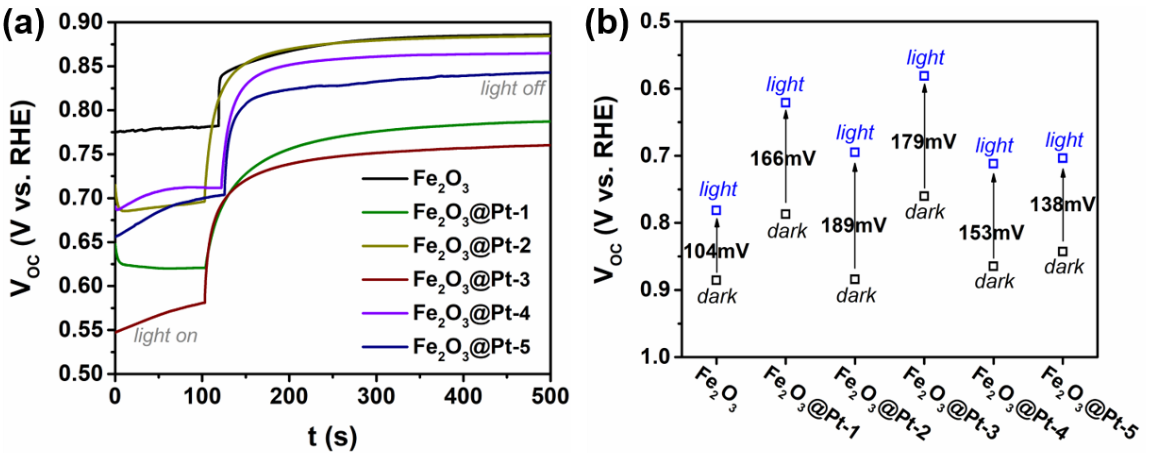

Figure S11 Open-circuit potential of Fe_2O_3 films with different concentrations of Pt NCs. (a) Photovoltages based on open-circuit potential test. (b) Open-circuit potential spectra under dark and illumination respectively.

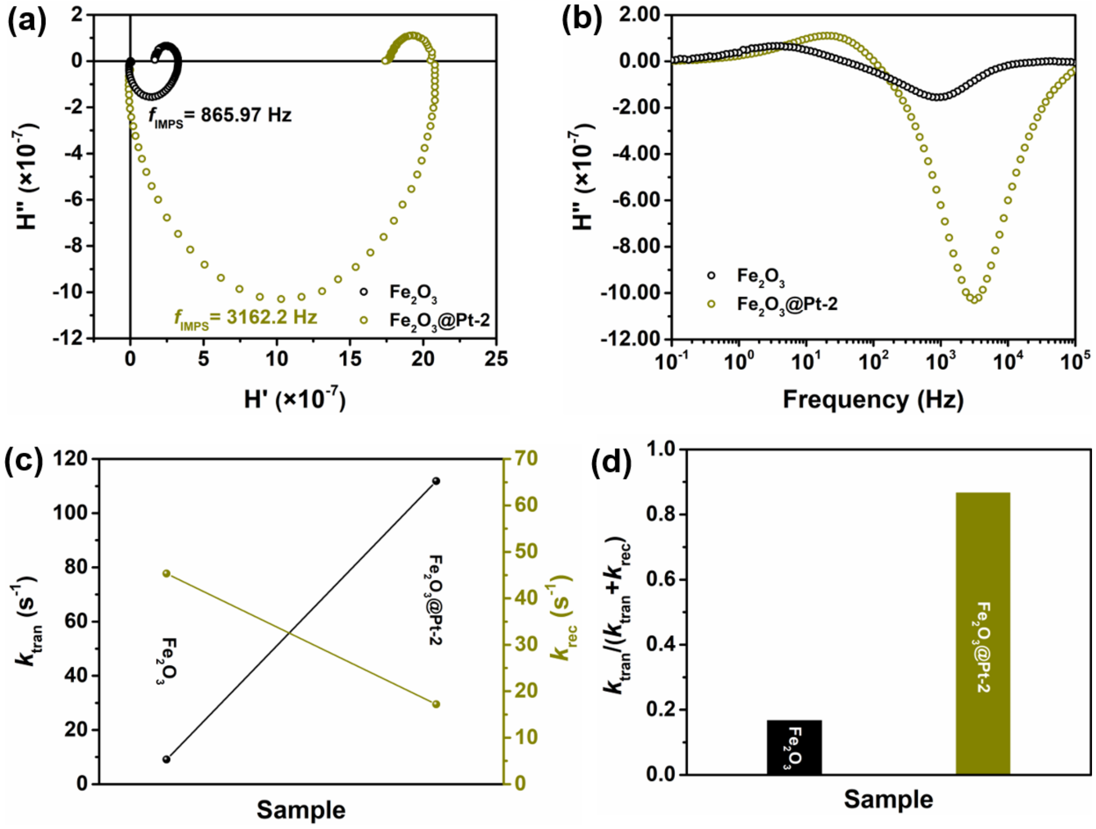

Figure S12 IMPS data of Fe_2O_3 and Fe_2O_3@Pt-2 films: (a) Nyquist plots. (b) Bode plots of the. (c) Rate constants derived from the dynamic model based on IMPS, including the charge-transfer constant (k_{tran}) and recombination constant (k_{rec}). (d) Charge transfer efficiency ($k_{tran}/(k_{tran}+k_{rec})$).

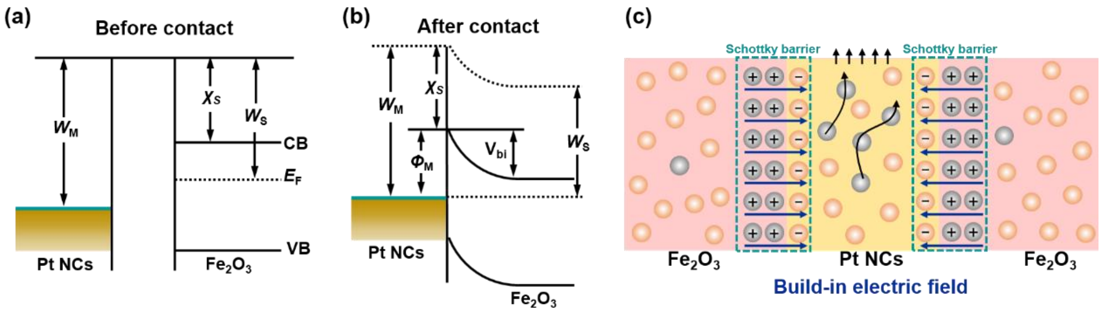

Figure S13 (a) Band gaps of Fe_2O_3 film and Pt nanocrystals before contact. (b) Band structure Fe_2O_3 film and Pt nanocrystals after contact. (c) Improved bulk charge separation of Fe_2O_3 film by embedding Pt nanocrystals in the Fe_2O_3 matrix.

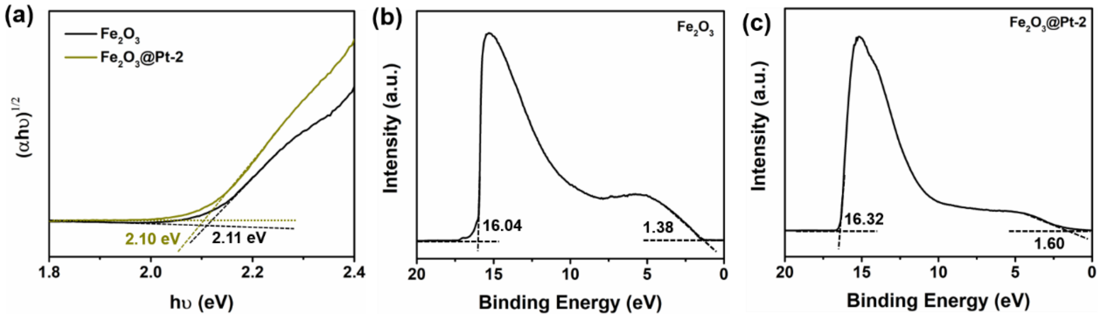

Figure S14 Band gaps and UPS spectra of Fe_2O_3 film and Fe_2O_3@Pt-2 film: (a) Band gaps of Fe_2O_3 and Fe_2O_3@Pt-2 film. UPS spectra of (b) Fe_2O_3 film, (c) Fe_2O_3@Pt-2 film.

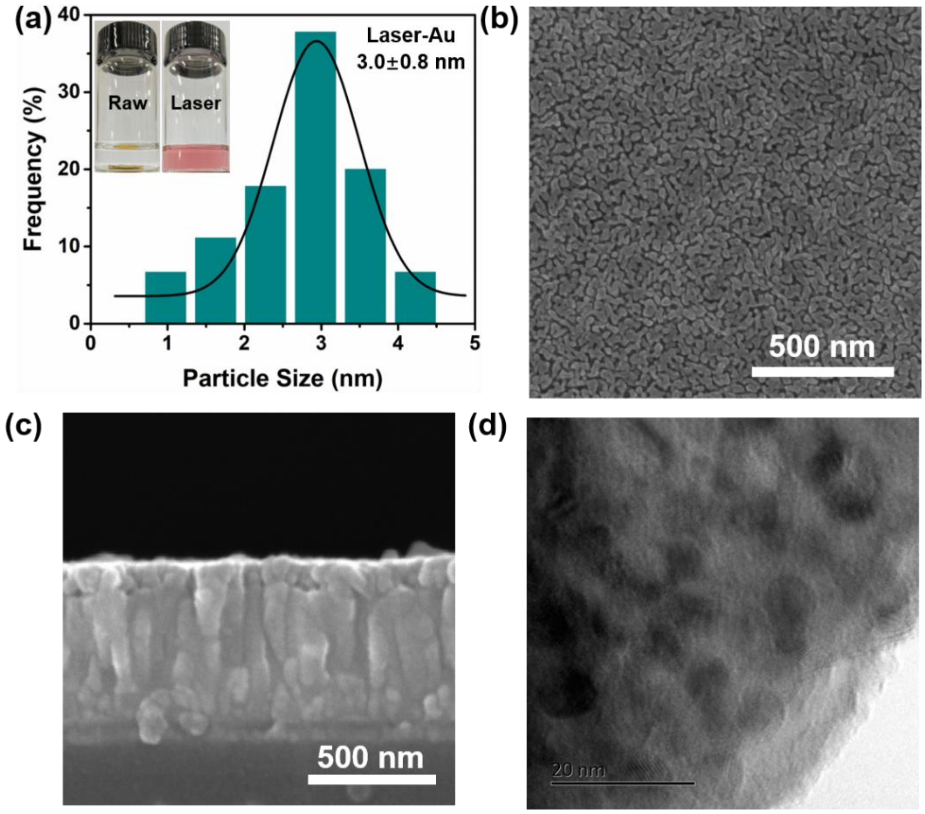

Figure S15 (a) Size distribution of Au nanocrystals (insert: photographs of Au solution before and after PLIL). (b) Surface SEM images of Fe_2O_3@Au film. (c) Cross-section SEM images of Fe_2O_3@Au film. (d) TEM image of Fe_2O_3@Au film.

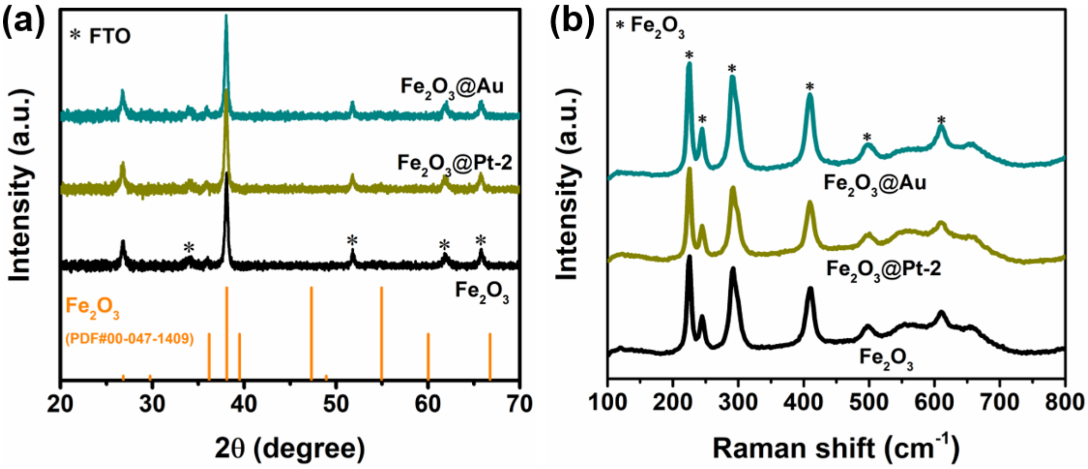

Figure S16 (a) XRD pattern, and (b) Raman spectra of Fe_2O_3, Fe_2O_3@Pt-2, and Fe_2O_3@Au films.

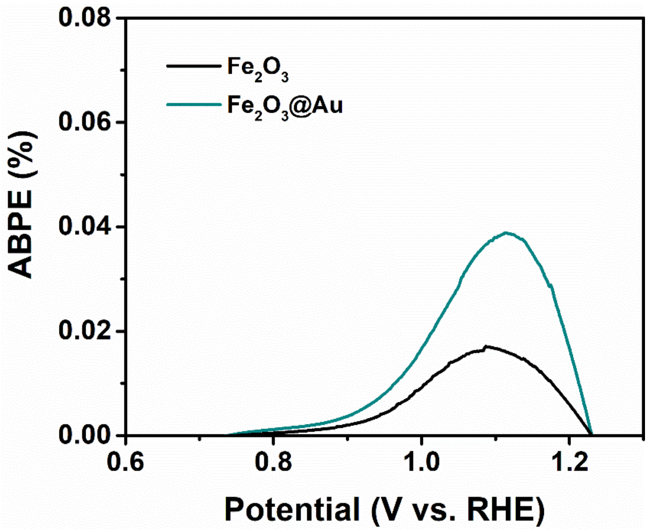

Figure S17 ABPE spectra of Fe_2O_3, and Fe_2O_3@Au films. ABPE spectra collected at the incident wavelength range from 300 to 700 nm at 1.23 V_{RHE} in 1M KOH electrolyte.

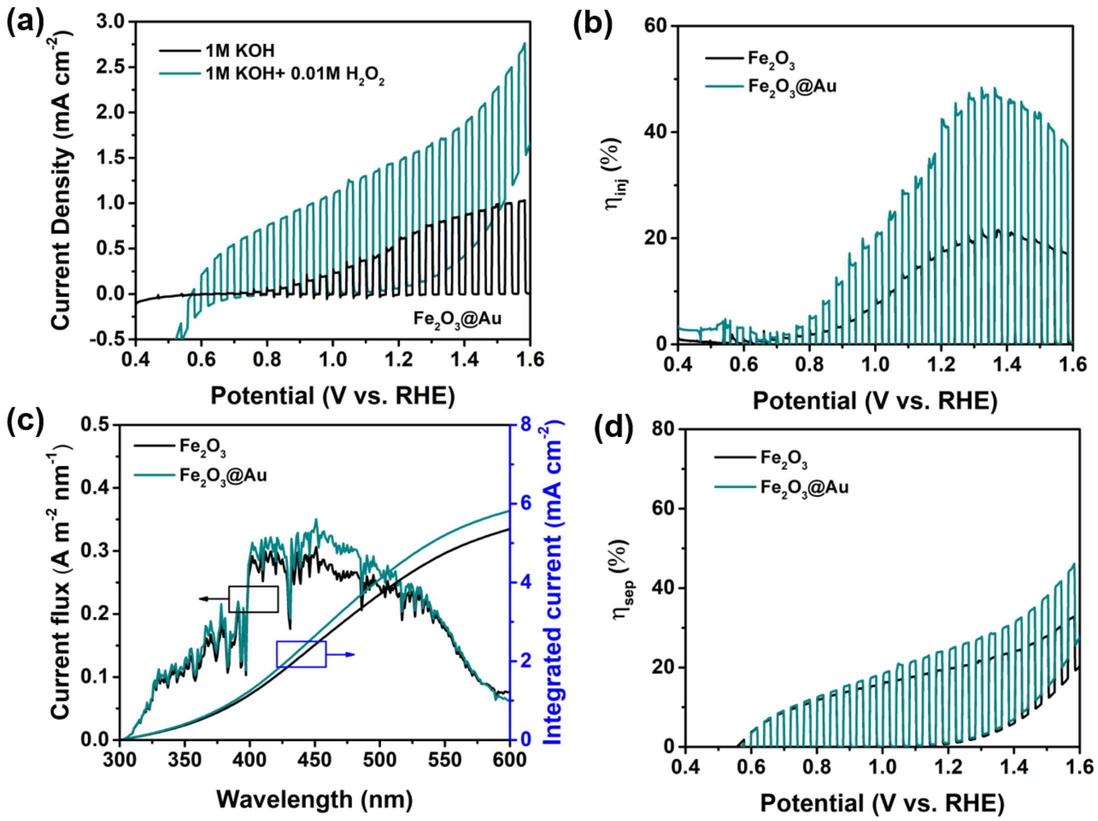

Figure S18 PEC performance of Fe_2O_3, and Fe_2O_3@Au films. (a) *J-V* curve in 1M KOH solution with 0.1 M H_2O_2 under AM 1.5G illumination (100 mW cm^{-2}). (b) Surface charge injection efficiency (η_{inj}). (c) The calculated current density flux and integrated current density (J_{abs}). (d) Bulk charge separation efficiency (η_{sep}).

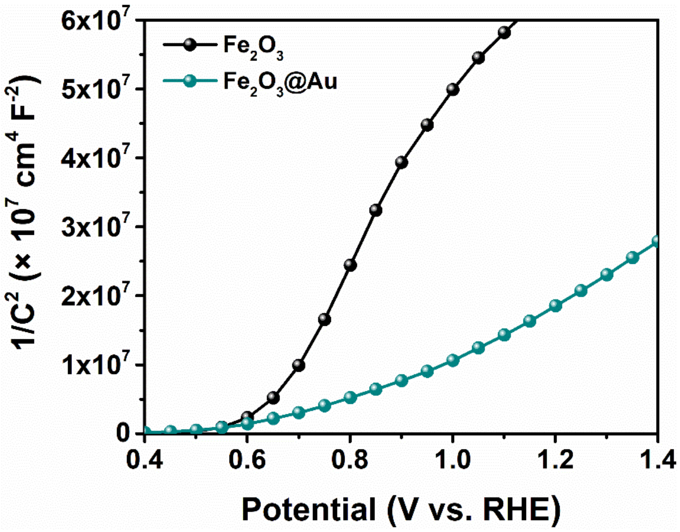

Figure S19 Mott-Schottky (M-S) plots measured in 1M KOH solution at 1 kHz frequency of Fe_2O_3, and Fe_2O_3@Au films.

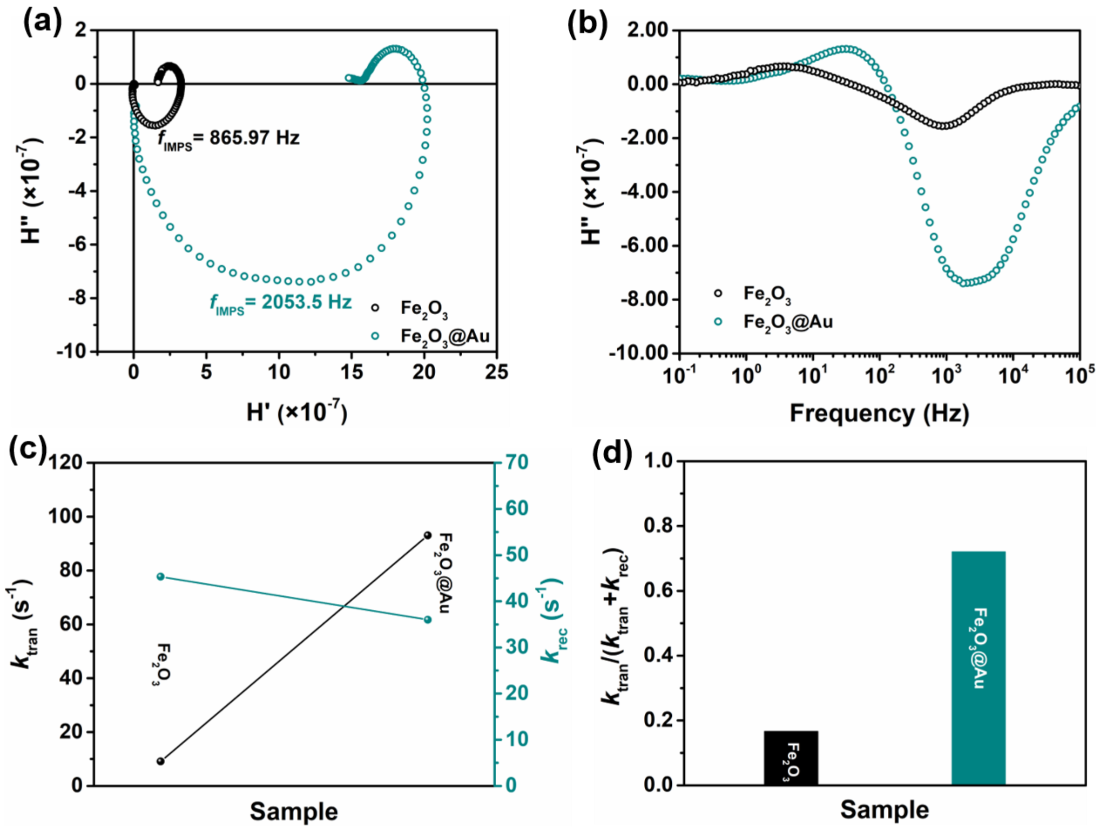

Figure S20 IMPS data of Fe_2O_3, and Fe_2O_3@Au films: (a) Nyquist plots. (b) Bode plots of the. (c) Rate constants derived from the dynamic model based on IMPS, including the charge-transfer constant (k_{tran}) and recombination constant (k_{rec}). (d) Charge transfer efficiency ($k_{tran}/(k_{tran}+k_{rec})$)

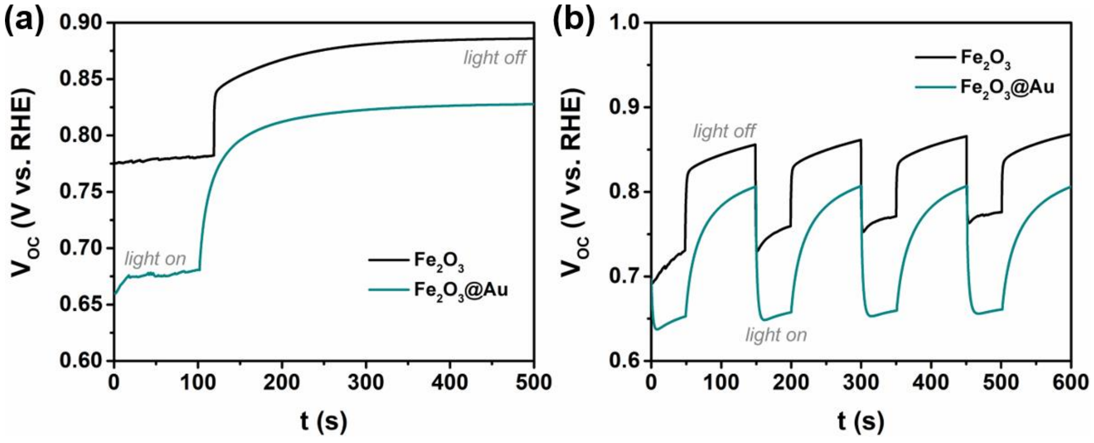

Figure S21 Open-circuit potential of Fe_2O_3, and Fe_2O_3@Au films. (a) Photovoltages based on open-circuit potential test. (b) Open-circuit potential spectra under dark and illumination respectively.

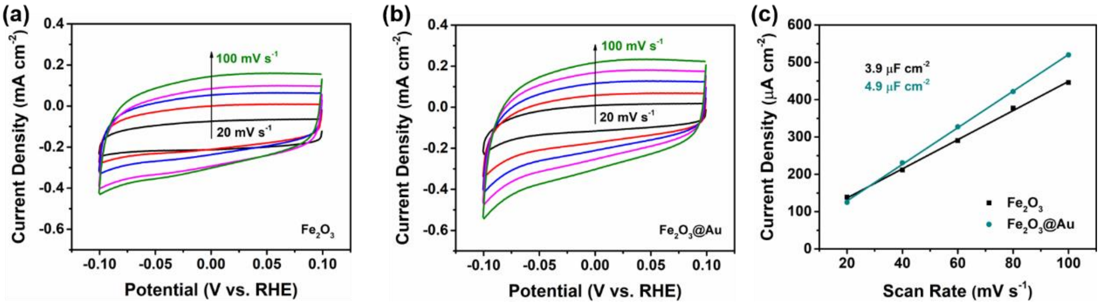

Figure S22 Typical cyclic voltammetry curves in 1M KOH of (a) Fe_2O_3 and (b) Fe_2O_3@Au films. (c) Estimate of the electrochemical active surface area of Fe_2O_3, and Fe_2O_3@Au films.

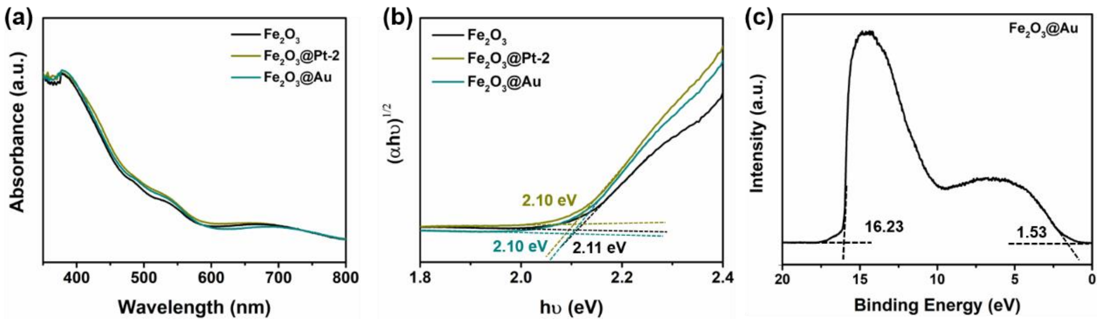

Figure S23 (a) UV-Vis spectra of Fe_2O_3, Fe_2O_3@Pt-2, and Fe_2O_3@Au films. (b) Band gaps of Fe_2O_3, Fe_2O_3@Pt-2, and Fe_2O_3@Au films. (c) UPS spectra of Fe_2O_3, Fe_2O_3@Pt-2, and Fe_2O_3@Au films.

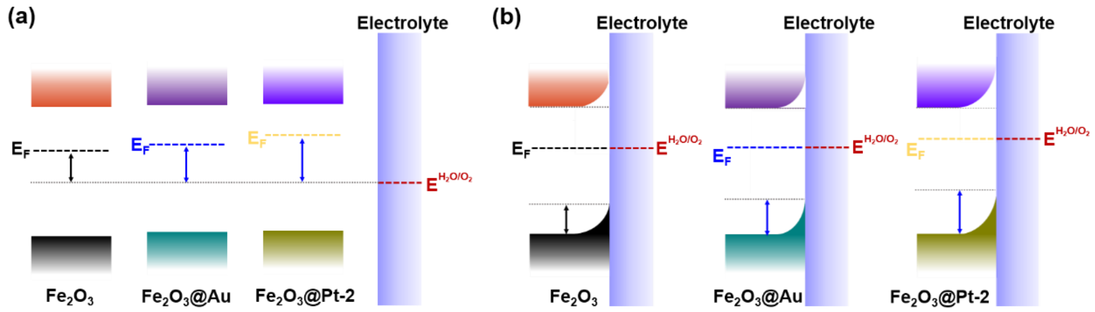

Figure S24 Schematic diagram of band structures of Fe_2O_3, Fe_2O_3@Pt-2, and Fe_2O_3@Au films: (a) Before and (b) after contacting with the electrolytes.

Table S1 The concentration of Pt and Au NCs colloidal solution determined by the ICP method.

Instrument model	ICP-OES: Agilent 5110					
Instrument parameter	Pump Rate	60r/min	Plasma gas	12.0L/min	Sample Flush Time	20s
	Nebulizer Flow	0.70L/min	Stable Time	20s	RF Power	1250w
	Auxiliary Gas	1.0L/min	Reading access time	5s		
Calculation method	C_1(mg/L)=C_0(mg/L)*f*V_0（mL）/V（mL）					
Sample	V（mL)	V_0（mL)	element	C_o（mg/L)	f	C_1（mg/L)
Pt NCs-1	2	10	Au	5.525	50	276.257015
Pt NCs-2	2	10	Au	5.526	50	276.2946125
Au NCs-1	2	10	Pt	2.284	50	114.219103
Au NCs-2	2	10	Pt	2.254	50	112.7001125

Table S2 The photoelectrochemical performance of Fe_2O_3, Fe_2O_3@Pt-2 and Fe_2O_3@Au films.

Samples	IPCE (%)	ABPE (%)
Fe_2O_3	4.53	0.017
Fe_2O_3@Pt-2	15.1	0.062
Fe_2O_3@Au	9.75	0.039

Table S3 Fitted parameters of the EIS of Fe_2O_3, Fe_2O_3@Pt-2 and Fe_2O_3@Au films.

Samples	R_{sol} (Ω cm^{-2})	R_{bulk} (Ω cm^{-2})	R_{ct} (Ω cm^{-2})
Fe_2O_3	24.2	76.5	8425
Fe_2O_3@Pt-2	20.3	44.8	619
Fe_2O_3@Au	22.1	48.7	706

Table S4 The photoelectrochemical performance of Fe_2O_3, Fe_2O_3@Pt-2 and Fe_2O_3@Au films.

Samples	J_{abs} (mA cm^{-2})	$J_{H_2O_2}$ (mA cm^{-2})	J_{H_2O} (mA cm^{-2})	η_{inj} (%)	η_{sep}(%)
Fe_2O_3	5.37	1.08	0.21	19.4	20.1
Fe_2O_3@Pt-2	5.99	2.40	1.16	48.3	40.1
Fe_2O_3@Au	5.84	1.52	0.62	40.8	26.0

Table S5 Under illumination or not and in 1M KOH solution, measured OCP values vs. RHE of Fe_2O_3 films with different concentrations of Pt NCs and Fe_2O_3@Au films.

Samples	OCV_{light} (mV)	OCV_{dark} (mV)	OCP (mV)
Fe_2O_3	781	885	104
Fe_2O_3@Pt-1	787	621	166
Fe_2O_3@Pt-2	695	884	189
Fe_2O_3@Pt-3	760	581	179
Fe_2O_3@Pt-4	865	712	153
Fe_2O_3@Pt-5	843	704	139
Fe_2O_3@Au	680	827	147

Table S6 Transfer time (τ_d) and charge transfer efficiency of photo-induced carriers generated by Fe_2O_3, Fe_2O_3@ Pt-2 and Fe_2O_3@Au films.

Samples	f_{IMPS} (Hz)	τ_d (ms)	k_{tran} (s^{-1})	k_{rec} (s^{-1})	$k_{tran}/(k_{tran} + k_{rec})$
Fe_2O_3	865.97	0.184	9.09	45.3	0.17
Fe_2O_3@Pt-2	3162.2	0.050	111.8	17.2	0.87
Fe_2O_3@Au	2053.5	0.078	93.0	36.0	0.72

Table S7 Band gap (E_g), VBM (VB), CBM (CB), and Fermi levels (E_F) of Fe_2O_3, Fe_2O_3@Pt-2, and Fe_2O_3@Au films.

Samples	E_g (eV)	VB (V_{RHE})	CB (V_{RHE})	E_F (V_{RHE})
Fe_2O_3	2.11	2.05	-0.06	0.67
Fe_2O_3@Pt-2	2.10	1.99	-0.11	0.48
Fe_2O_3@Au	2.10	2.01	-0.09	0.39

The work function is defined as the difference value between the vacuum energy level and Fermi level, which can be calculated from the low kinetic energy cutoff in the secondary emission feature. The photon energy of the UV source (He I discharge) is 21.21 eV. Given that the Fermi level at the surface of Fe_2O_3 films are independent and the work functions are determined to be 5.17, 4.98, and 4.89 eV for Fe_2O_3, Fe_2O_3@Pt-2, and Fe_2O_3@Au, respectively. Using 4.5 eV vs. vacuum as the reference value for the electrochemical reduction of water (0.0 V_{RHE}), Fermi levels of Fe_2O_3, Fe_2O_3@Pt-2, and Fe_2O_3@Au are located in 0.67, 0.48, and 0.39 V_{RHE}. The positions of the VBM were estimated to be 2.05, 1.99, and 2.01 V_{RHE} by magnified UPS spectra in Figures S13b-c and S22c. Using Fermi level and VBM, along with the measured bandgap (Figures S13a and S22b), the positions of the CBM were estimated to be -0.06, -0.11, and -0.09 V_{RHE}. As commented by the reviewer, we have added more details of the band structure of Fe_2O_3 and Fe_2O_3@Pt-2 films.

References

1 Woo Kim Tae, Kyoung-Shin Choi. Nanoporous $BiVO_4$ Photoanodes with Dual-Layer Oxygen Evolution Catalysts for Solar Water Splitting. *Science.* 2014;343(6174):990-994. doi: 10.1126/science.1246913.

2 Chengcheng Li, Tuo Wang, Zhibin Luo, Shanshan Liu, Jinlong Gong. Enhanced Charge Separation through ALD-Modified Fe_2O_3/Fe_2TiO_5 Nanorod Heterojunction for Photoelectrochemical Water Oxidation. *Small.* 2016;12(25):3415-3422. doi: 10.1002/smll.201600940.

3 Sha-Sha Yi, Jun-Min Yan, Qing Jiang. Carbon quantum dot sensitized integrated Fe_2O_3@g-C_3N_4 core–shell nanoarray photoanode towards highly efficient water oxidation. *Journal of Materials Chemistry A.* 2018;6(21):9839-9845. doi: 10.1039/C8TA01908H.

4 Qiang Rui, Lei Wang, Yajun Zhang, Chenchen Feng, Beibei Zhang, Shurong Fu, Huilin Guo, Hongyan Hu, Yingpu Bi. Synergistic effects of P-doping and a MnO_2 cocatalyst on Fe_2O_3 nanorod photoanodes for efficient solar water splitting. *Journal of Materials Chemistry A.* 2018;6(16):7021-7026. doi: https://doi.org/10.1039/C8TA00556G.

5 Ludmilla Steier, Jingshan Luo, Marcel Schreier, Matthew T. Mayer, Timo Sajavaara, Michael Grätzel. Low-Temperature Atomic Layer Deposition of Crystalline and Photoactive Ultrathin Hematite Films for Solar Water Splitting. *ACS Nano.* 2015;9(12):11775-11783. doi: https://doi.org/10.1021/acsnano. 5b03694.

6 Roel van de Krol, Michael Grätzel. Photoelectrochemical Hydrogen Production. London: *Springer*. 2012.

7 Haiyan Jin, Shanjun Mao, Guopeng Zhan, Fan Xu, Xiaobing Bao, Yong Wang. Fe incorporated α-$Co(OH)_2$ nanosheets with remarkably improved activity towards the oxygen evolution reaction. *Journal of Materials Chemistry A.* 2017;5(3):1078-1084. doi: 10.1039/C6TA09959A.

8 Feng Li, Jing Li, Lili Gao, Yiping Hu, Xuefeng Long, Shenqi Wei, Chenglong Wang, Jun Jin, Jiantai Ma. Construction of an efficient hole migration pathway on hematite for efficient photoelectrochemical water oxidation. *Journal of Materials Chemistry A.* 2018;6(46):23478-23485. doi: 10.1039/C8TA07832G.

9 Haiqing Ma, Jun Beom Hwang, Weon Sik Chae, Hee Suk Chung, Sun Hee Choi, Mahadeo A. Mahadik, Hyun Hwi Lee, Jum Suk Jang. Magnetron sputtering strategy for Zr-Fe_2O_3

nanorod photoanode fabricated from ZrO_x/β-FeOOH nanorods for photoelectrochemical water splitting. *Applied Surface Science.* 2021;549(149233. doi: https://doi.org/10.1016/j.apsusc.2021.149233.

10 Carolin Zachäus, Fatwa F. Abdi, Laurence M. Peter, Roel van de Krol. Photocurrent of $BiVO_4$ is limited by surface recombination, not surface catalysis. *Chemical Science.* 2017;8(5):3712-3719. doi: https://doi.org/10.1039/ C7SC00363C.

11 Xingming Ning, Yali Wu, Xiaofang Ma, Zhen Zhang, Ruiqin Gao, Jing Chen, Duoliang Shan, Xiaoquan Lu. A Novel Charge Transfer Channel to Simultaneously Enhance Photocatalytic Water Splitting Activity and Stability of CdS. *Advanced Functional Materials.* 2019;29(40):1902992. doi: 10.1002/adfm.201902992.
